# Association of serum 25-hydroxyvitamin D concentrations with all-cause and cardiovascular mortality among US adults with prehypertension: a prospective cohort study

**DOI:** 10.1186/s41043-024-00515-5

**Published:** 2024-02-06

**Authors:** Yongmei Zhou, Yu Chen, Fuli Chen, Gang Li, Long Zhou

**Affiliations:** 1 Department of Cardiology, Sichuan Provincial People’s Hospital, University of Electronic Science and Technology of China, Chengdu, China; 2 Institute of Cardiovascular Diseases and Department of Cardiology, Sichuan Provincial People’s Hospital, School of Medicine, University of Electronic Science and Technology of China, Chengdu, China

**Keywords:** Serum 25-hydroxyvitamin D, All-cause mortality, CVD mortality, Prehypertension, Cohort study

## Abstract

**Background:**

Prehypertension affects 25–50% of adults worldwide and no prior study has examined the relationship between serum 25-hydroxyvitamin D [25(OH)D] concentrations and mortality risk in individuals with prehypertension. This study aims to investigate the association of serum 25(OH)D concentrations with all-cause and CVD mortality among prehypertensive adults by utilizing data from the US National Health and Nutrition Examination Survey (NHANES) 2007–2014 and linked 2019 mortality file.

**Methods:**

We included 4345 prehypertensive adults who participated in the NHANES between 2007 and 2014 and were followed up until 31 December 2019. Weighted Cox proportional hazards models were used with adjustments for multiple covariates to calculate the hazard ratio (HR) and 95% confidence interval (CI) for the risks of dying from any cause and CVD.

**Results:**

During a median follow-up of 8.8 years, 335 deaths from any causes were documented, of which 88 participants died from CVD. Compared with participants with sufficient 25(OH)D (≥ 75 nmol/L), the multivariate-adjusted HRs and 95% CIs for participants with severe deficiency (< 25 nmol/L), moderate deficiency (25–49.9 nmol/L), and insufficient concentrations (50–74.9 nmol/L) of serum 25(OH)D for all-cause death were 2.83 (1.46–5.52), 1.17 (0.74–1.86), and 1.36 (0.93–1.98), respectively. Similarly, the multivariable-adjusted HRs and 95%CIs for CVD death were 4.14 (1.10–15.51), 1.23 (0.46–3.28), and 1.73 (0.96–3.14), respectively. We found that there was a 9% reduction in the risk of death from all causes and a 14% reduction in the risk of death from CVD for every 10 nmol/L increase in serum 25(OH)D concentrations.

**Conclusion:**

Severe serum 25(OH)D deficiency among prehypertensive adults was associated with increased risk of mortality from all causes as well as from CVD. Our work suggests that supplementing with vitamin D may prevent premature death in severely deficient individuals with prehypertension.

**Supplementary Information:**

The online version contains supplementary material available at 10.1186/s41043-024-00515-5.

## Introduction

As a steroid hormone, vitamin D plays an essential role in bone metabolism for its function in the regulation of calcium and phosphate [1]. The role of vitamin D beyond the bone metabolism has received a lot of attention in recent years [2]. The link between vitamin D deficiency and an increased risk of hypertension has continuously been demonstrated by meta-analyses of prospective cohort studies and cross-sectional investigations [3, 4]. Several cohort studies based on the US National Health and Nutrition Examination Survey (NHANES) have also shown that serum 25-hydroxyvitamin D [25(OH)D] concentrations are related to the risk of cardiovascular and all-cause mortality in both the general population and in individuals with specific conditions like diabetes, hyperlipidemia, and non-alcoholic fatty liver disease (NAFLD) [5–8]. In US general population, Ford et al. found that concentrations of vitamin D were weakly and inversely related to all-cause mortality after adjustment for age, sex, ethnicity, education level, smoking status, drinking status, physical activity, vitamin supplement use, systolic blood pressure, serum lipids, hemoglobin A1c, waist circumference, and history of cardiovascular disease (CVD) [5]. Wan et al. found that higher serum 25(OH)D levels were significantly and linearly associated with lower all-cause and CVD mortality in individuals with diabetes. There was a 31% reduced risk of all-cause mortality and a 38% reduced risk of CVD mortality per one-unit increment in natural log-transformed 25(OH)D [6]. Chen et al. reported that lower serum 25(OH)D than 25.6 and 25.2 ng/mL were, respectively, associated with a gradual increase in a risk for all-cause and cardiovascular mortality in patients with hyperlipidemia [7]. Zhang et al. reported that compared with participants with serum 25(OH)D concentrations ≤ 30.0 nmol/L, the multivariable-adjusted hazard ratios (HRs) and 95% confidence intervals (CIs) of all-cause mortality were 0.63 (0.42, 0.95) for participants with NAFLD having serum 25(OH)D > 75.0 nmol/L [8]. In addition, two prospective cohort studies evaluated the association between serum 25(OH)D and mortality risk in individuals with hypertension. Zhao et al. analyzed data from the US NHANES and found that serum 25(OH)D were linearly and inversely associated with the risk of mortality from all causes (HR:0.97, 95%CI 0.95–0.99) and from CVD (HR:0.95, 95%CI 0.91–0.99) after adjustment for age, sex, ethnicity, education, body mass index (BMI), smoking status, drinking status, physical activity, vitamin supplement use, serum lipids, and history of CVD [9]. Based on the Korean National Health and Nutrition Examination Survey (KNHANES), Park et al. found that serum 25(OH)D below 20 ng/mL is associated with a higher risk of mortality in Korean adults with hypertension after adjusting for age, sex, region, income, smoking status, alcohol consumption, physical activity, and BMI [10]. However, long-term effects of serum 25(OH)D on the risk of death from all causes and CVD are less well understood in individuals with prehypertension. The 7th Joint National Committee on Prevention, Detection, Evaluation, and Treatment of High Blood Pressure (JNC 7) first coined the term "prehypertension," which is defined as having systolic blood pressure (SBP) readings between 120 and 139 mmHg and diastolic blood pressure (DBP) readings between 80 and 89 mmHg [11]. Prehypertension is not a disease, but a condition that exists between normal blood pressure and hypertension [12]. Prehypertension, which affects between 25 and 50 percent of adults worldwide, has been confirmed in studies to be a strong risk factor for hypertension and CVD [13]. Therefore, determining modifiable risk factors is crucial to preventing the emergence of adverse consequences in prehypertensive individuals. Based on findings from previous studies, we assume that serum 25(OH)D levels are inversely associated with the risk of all-cause mortality and CVD mortality in individuals with prehypertension. The current study aims to use data from the NHANES 2007–2014 and linked 2019 mortality file to examine the association of serum 25(OH)D concentrations with all-cause and CVD death among individuals with prehypertension.

## Methods

### Study population

**Fig. 1:**
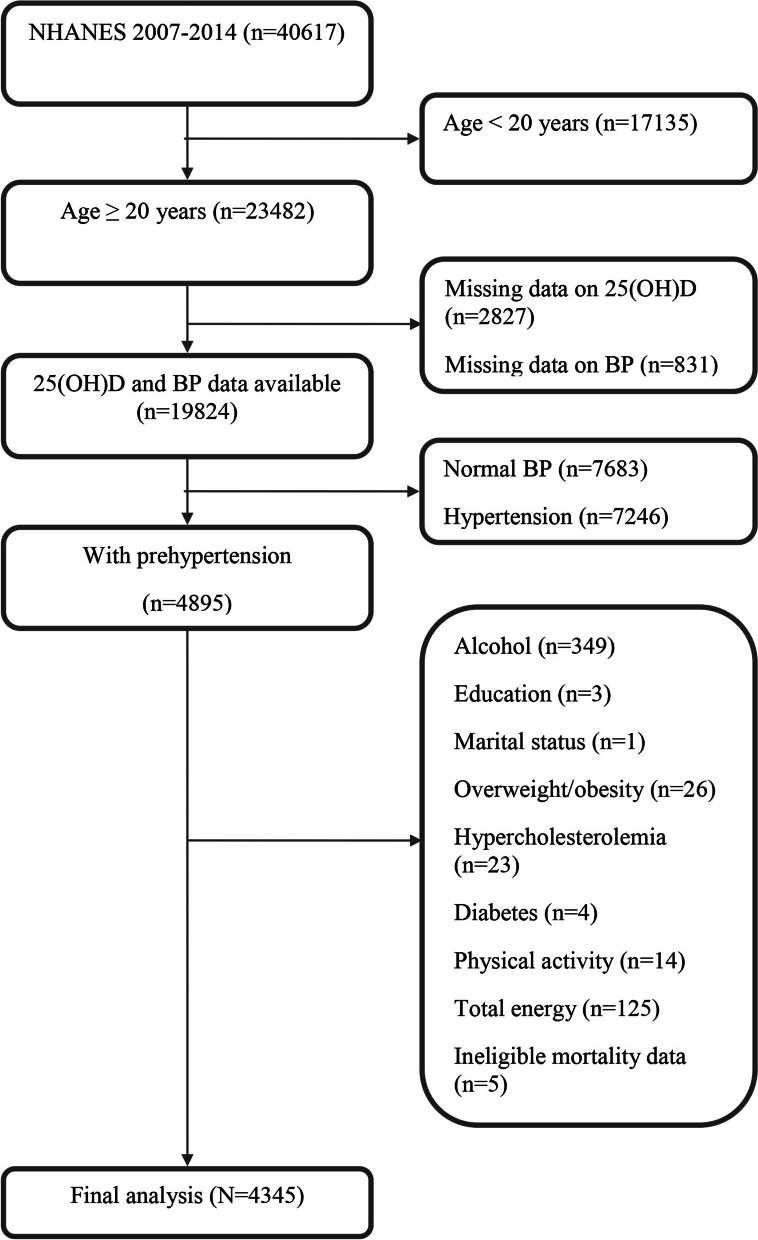
Flow chart of participant selection

The study population was derived from NHANES, which was a program of studies designed to evaluate the health and nutritional status of adults and children in the US. In our earlier investigations, the population of the NHANES study and the sample design were explained in detail [14, 15]. NHANES enrolled a total of 40,617 participants in the four consecutive 2-year cycles. At baseline, there were 10,149 participants in NHANES 2007–2008, 10,537 in NHANES 2009–2010, 9756 in NHANES 2011–2012, and 10,175 in NHANES 2013–2014. Of the 40,617 participants available, we focused on the 23,482 individuals who were 20 years of age and older in this study and applied specific exclusion criteria. We firstly excluded 2827 participants with missing serum 25(OH)D data and 831 participants with missing BP information. We also excluded 7683 participants whose BP was normal (SBP < 120 mmHg and DBP < 80 mmHg and with no history of antihypertensive medication use) and 7246 patients who were hypertensive (defined as SBP ≥ 140 mmHg or DBP ≥ 90 mmHg or with a history of antihypertensive medication use). We additionally excluded 545 participants with missing data on covariates (349 on alcohol intake, 3 on education, 1 on marital status, 26 on overweight/obesity, 14 on physical activity, 125 on energy intake, 4 on diabetes, and 23 on hypercholesterolemia), and 5 participants whose mortality data were ineligible. We finally included 4345 individuals with prehypertension in our analysis. Figure 1 displays a flowchart detailing the procedure for choosing participants. All participants supplied written informed consent. The National Center for Health Statistics (NCHS) institutional review board gave its approval to the NHANES protocol in Protocol #2011-17.

### Baseline data collection

Age, sex, race/ethnicity (Hispanic, White, Black, and other races), educational attainment (< high school, high school, and ≥ high school), and marital status (never married, married, cohabiting, separated, widowed, and divorced) are all considered demographic factors. The categories of married and cohabiting were combined to simplify analysis. The categories of divorced, widowed, and separated were also combined for the same reason. As a lifestyle factor, smoking status was divided into three categories: current, former, and never. Participants who had consumed alcohol at least 12 times in the previous year were considered drinkers. Time spent in sedentary activity (hours/d) is defined as all time spent seated, excluding time spent sleeping, and is a reflection of physical activity in this study.

Dietary intake was collected using two 24-h dietary recall interviews by trained staff. The first dietary recall interview was collected face-to-face in the Medical Examinatin Center, and the second was collected by telephone 3 to 10 days later. Dietary intake was estimated by the mean of the two dietary recalls. Dietary energy (in kcal/d), sodium (in mg/d), potassium (in mg/d) intakes, and vitamin D supplement use were used as the covariates.

A trained staff member used standardized tools to measure each subject's weight (in kg) and height (in cm). Body mass index (BMI) was determined based on height and weight as follows: weight in kilograms divided by square of height in meters. In accordance with the guidelines of the World Health Organization Expert Consultation, we adopted BMI ≥ 25 kg/m^2^ to define overweight/obesity [16]. After resting quietly in a seated position for 5 min, BP was measured using a mercury sphygmomanometer three times. The mean of three readings of BP was used in the analysis. Prehypertension is defined as having SBP readings between 120 and 139 mmHg and DBP readings between 80 and 89 mmHg and never used antihypertensive medication [11].

Serum specimens were processed, stored, and shipped to the Division of Environmental Health Laboratory Sciences, National Center for Environmental Health, Centers for Disease Control and Prevention for analysis. On a Roche/Hitachi Cobas 6000 Analyzer (Roche Diagnostics, Indianapolis, IN.), total cholesterol (TC) was measured using an enzymatic method, and hypercholesterolemia was defined as TC ≥ 6.2 mmol/L or current medication use [17]. Hemoglobin A1c was measured on the Tosoh Automated Glycohemoglobin Analyzer HLC-723G8 (Tosoh Bioscience, Inc., South San Francisco, CA.) and diabetes was defined as hemoglobin A1c ≥ 6.5% and/or continued usage of insulin or hypoglycemic medications [18]. Comorbidities at baseline included hypercholesterolemia, diabetes, and a history of physician-diagnosed heart failure (HF), coronary heart disease (CHD), stroke, and cancer.

Beginning with the NHANES 2007–2008 cycle, serum 25(OH)D concentrations were measured using a standardized liquid chromatography–tandem mass spectrometry (LC–MS/MS) approach [19]. We classified serum 25(OH)D concentrations into four categories: severe deficiency (< 25 nmol/L), moderate deficiency (25–49.9 nmol/L), insufficient (50–74.9 nmol/L), and sufficient (≥ 75 nmol/L) according to earlier studies [6, 19].

### Assessment of mortality

The National Center for Health Statistics (NCHS) gathered mortality statistics by linking it to the National Death Index (NDI) up to 31 December 2019. The 2019 NCHS Public-Use Linked Mortality Files were used to generate mortality information for all included participants, including mortality status, cause of death, and follow-up duration. Cause-specific death was determined using the International Classification of Diseases, 10th Revision (ICD-10) system. For the purposes of this study, deaths from any cause were referred to as "all-cause deaths", whereas deaths from cardiovascular disease (ICD-10 codes I00-I09, I11, I13, and I20-I51) or cerebrovascular disease (ICD-10 codes I60-I69) were referred to as "CVD deaths".

### Statistical analysis

Data are presented as weighted mean ± standard deviation (SD) for continuous variables and as n (weighted percentage) for categorical variables according to NHANES analytic guidelines. To compare the between-group differences in characteristics, we performed chi-square tests for categorical variables and one-way ANOVA for continuous data. In order to decrease the false discovery rate and avoid type I errors, adjusted p-values were assessed by using the Bonferroni method [20]. Weighted Cox proportional hazards models were used with adjusting for demographic factors (age, sex, ethnicity, education levels, and marital status), lifestyle factors (smoking status, drinking status, sedentary hours), nutritional factors (overweight/obesity, total energy intake, sodium intake, potassium, and vitamin D supplement use), and comorbidities (diabetes, hypercholesterolemia, HF, CHD, stroke, and cancer). We used the group of "sufficient" as the reference to determine the HR and 95% CI for the risks of dying from any cause and CVD. Serum 25(OH)D was also analyzed as a continuous variable. The dose–response analysis was conducted using the restricted cubic spline model after accounting for the aforementioned confounding factors. In order to further explore the relationships between serum 25(OH)D and the risk of all-cause and CVD mortality, we also conducted a number of subgroup analyses based on baseline characteristics and the interactions between serum 25(OH)D and baseline characteristics were tested. Statistical significance was defined as a two-tailed p value < 0.05. All statistical analyses were performed using SAS version 9.4 (SAS Institute, Cary, NC, USA). The restricted cubic spline curves were generated using R 3.6.1 (R Foundation for Statistical Computing, Vienna, Austria).

## Results

The study included 4345 prehypertensive adults in total. The median (IQR) age of all participants was 46.0 (34.0–59.0) years, and the median (IQR) serum 25(OH)D was 60.2 (44.1–76.9) nmol/L. The baseline characteristics of the study population are shown in Table 1. Compared with individuals with sufficient serum 25(OH)D, those with severe deficiency of serum 25(OH)D were younger, more likely to be men, of the black race, to have less schooling, to have never been married, to be current smoker, and to be overweight or obesity. However, compared to individuals with sufficient serum 25(OH)D concentrations, those with severe serum 25(OH)D deficiency were less likely to drink, to have hypercholesterolemia, and to use vitamin D supplement.

**Table 1:** Characteristics of the study population according to serum 25-hydroxyvitamin D concentrations (n = 4345)^†^

Characteristics	Severe deficiency	Moderate deficiency	Insufficient	Sufficient	p-values	Adjusted p-values^‡^
Serum 25(OH)D concentrations	< 25 nmol/L	25–49.9 nmol/L	50–74.9 nmol/L	≥ 75 nmol/L		
Sample size	201	1245	1694	1205		
Age, years	38.7 ± 15.2	42.6 ± 15.5	44.9 ± 15.6	49.5 ± 16.7	< 0.0001	0.0018
Men, n (%)	115 (56.2)	757 (59.8)	1124 (66.0)	677 (55.3)	< 0.0001	0.0018
Ethnicity, n (%)					< 0.0001	0.0018
Hispanic	39 (18.5)	382 (21.5)	519 (16.6)	180 (5.7)		
White	25 (19.4)	330 (47.6)	800 (70.8)	850 (87.4)		
Black	115 (50.4)	401 (22.6)	227 (6.6)	83 (2.5)		
Other	22 (11.7)	132 (8.3)	148 (6.0)	92 (4.5)		
Education, n (%)					< 0.0001	0.0018
Less than high school	53 (21.9)	334 (20.6)	457 (18.1)	221 (12.6)		
High school	56 (29.3)	317 (25.3)	365 (21.9)	260 (20.8)		
More than high school	92 (48.8)	594 (54.1)	872 (60.0)	724 (66.6)		
Marital status, n (%)					< 0.0001	0.0018
Married	81 (40.5)	685 (55.9)	1102 (66.2)	789 (69.4)		
Separated	42 (20.1)	237 (17.8)	297 (15.6)	250 (16.7)		
Never married	78 (39.4)	323 (26.3)	295 (18.3)	166 (13.9)		
Smoking status, n (%)					< 0.0001	0.0018
Current	68 (36.4)	333 (26.4)	373 (22.5)	233 (18.5)		
Former	25 (10.2)	220 (17.6)	426 (24.3)	335 (28.4)		
Never	108 (53.5)	692 (56.0)	895 (53.2)	637 (53.1)		
Drinker, n (%)	141 (73.4)	937 (78.9)	1313 (81.6)	948 (84.3)	< 0.0001	0.0018
Overweight/obesity, n (%)	148 (71.3)	963 (78.7)	1315 (79.1)	773 (66.1)	< 0.0001	0.0018
Diabetes, n (%)	15 (5.9)	169 (10.7)	151 (6.7)	88 (4.9)	< 0.0001	0.0018
Hypercholesterolemia, n (%)	23 (12.6)	256 (19.8)	398 (24.5)	381 (31.1)	< 0.0001	0.0018
Heart failure, n (%)	4 (1.4)	21 (1.7)	16 (0.8)	20 (1.1)	< 0.0001	0.0018
Coronary heart disease, n (%)	3 (1.1)	20 (1.2)	29 (1.7)	32 (1.6)	< 0.0001	0.0018
Stroke, n (%)	2 (0.7)	18 (1.5)	21 (1.0)	26 (1.5)	< 0.0001	0.0018
Cancer, n (%)	6 (4.7)	49 (4.1)	118 (7.2)	152 (11.9)	< 0.0001	0.0018
Sedentary activity, hours/d	6.5 ± 3.2	6.3 ± 3.5	6.0 ± 3.3	5.9 ± 3.3	0.0064	0.1152
Total energy intake, kcal/d	2116.7 ± 1057.2	2225.0 ± 947.1	2278.8 ± 874.8	2263.0 ± 967.4	0.1631	0.1631
Sodium intake, mg/d	3424.0 ± 1713.1	3718.9 ± 1725.0	3736.4 ± 1593.7	3651.3 ± 1706.6	0.1159	0.1227
Potassium intake, mg/d	2375.0 ± 1103.0	2563.7 ± 1063.9	2790.0 ± 1088.9	2975.1 ± 1189.5	< 0.0001	0.0018
Vitamin D supplement use, n (%)	17 (11.3)	157 (12.5)	488 (28.3)	629 (52.1)	< 0.0001	0.0018

^†^Values are presented as weighted mean ± SD or frequency (weighted percentage) when appropriate

^‡^Adjusted p-values were assessed by bonferroni method

Table 2 shows the relationships between serum 25(OH)D and the risk of all-cause and CVD mortality. The multivariable-adjusted HRs and 95% CIs for all-cause mortality were 2.83 (1.46–5.52), 1.17 (0.74–1.86), and 1.36 (0.93–1.98) for the groups with severe deficiency, moderate deficiency, and insufficient concentrations of serum 25(OH)D, respectively. Similarly, the multivariable-adjusted HRs and 95%CIs for CVD mortality were 4.14 (1.10–15.51), 1.23 (0.46–3.28), and 1.73 (0.96–3.14), respectively. When serum 25(OH)D was considered a continuous variable, we observed that the risk of both CVD mortality (HR: 0.86, 95%CI 0.76–0.98) and all-cause mortality (HR: 0.91, 95%CI 0.85–0.97) decreased with each 10 nmol/L increase in serum 25(OH)D concentrations.

**Table 2:** Hazard ratios and 95% confidence intervals for all-cause and cardiovascular disease mortality according to serum 25(OH)D concentration in individuals with prehypertension^†^

	No. of death (%)	Hazard ratios and 95% confidence intervals‡		
		Model 1	Model 2	Model 3
**All-cause mortality**				
Categorical serum 25(OH)D concentration				
Severe deficiency	18 (9.0)	0.87 (0.41–1.88)	2.58 (1.31–5.05)	2.83 (1.46–5.52)
Moderate deficiency	82 (6.6)	0.63 (0.38–1.04)	1.11 (0.68–1.82)	1.17 (0.74–1.86)
Insufficient	122 (7.2)	0.80 (0.50–1.26)	1.30 (0.89–1.88)	1.36 (0.93–1.98)
Sufficient	113 (9.4)	1 (Ref)	1 (Ref)	1 (Ref)
Continuous serum 25(OH)D concentration, per 10 nmol/L increase		1.04 (0.98–1.10)	0.92 (0.86–0.98)	0.91 (0.85–0.97)
**CVD mortality**				
Categorical serum 25(OH)D				
Severe deficiency	6 (3.0)	0.49 (0.09–2.63)	3.10 (0.87–11.05)	4.14 (1.10–15.51)
Moderate deficiency	18 (1.5)	0.49 (0.13–0.82)	1.22 (0.44–3.39)	1.23 (0.46–3.28)
Insufficient	34 (2.0)	0.78 (0.25–2.49)	1.53 (0.83–2.80)	1.73 (0.96–3.14)
Sufficient	30 (2.5)	1 (Ref)	1 (Ref)	1 (Ref)
Continuous serum 25(OH)D, per 10 nmol/L increase		1.07 (0.94–1.21)	0.87 (0.76–0.98)	0.86 (0.76–0.98)

^†^Hazard ratios and 95% confidence intervals were calculated by weighted Cox proportional hazards models. CVD, cardiovascular disease; 25(OH)D, 25-hydroxyvitamin D

^‡^Model 1 was unadjusted. Model 2 adjusted for age, sex, ethnicity, education level, marital status, smoking status, drinking status, sedentary activity, total energy intake, sodium intake, potassium intake, overweight/obesity, and vitamin D supplement use. Model 3 adjusted for variables included in Model 2 plus comorbidities such as diabetes, hypercholesterolemia, heart failure, coronary heart disease, stroke, and cancer

**Fig. 2:**
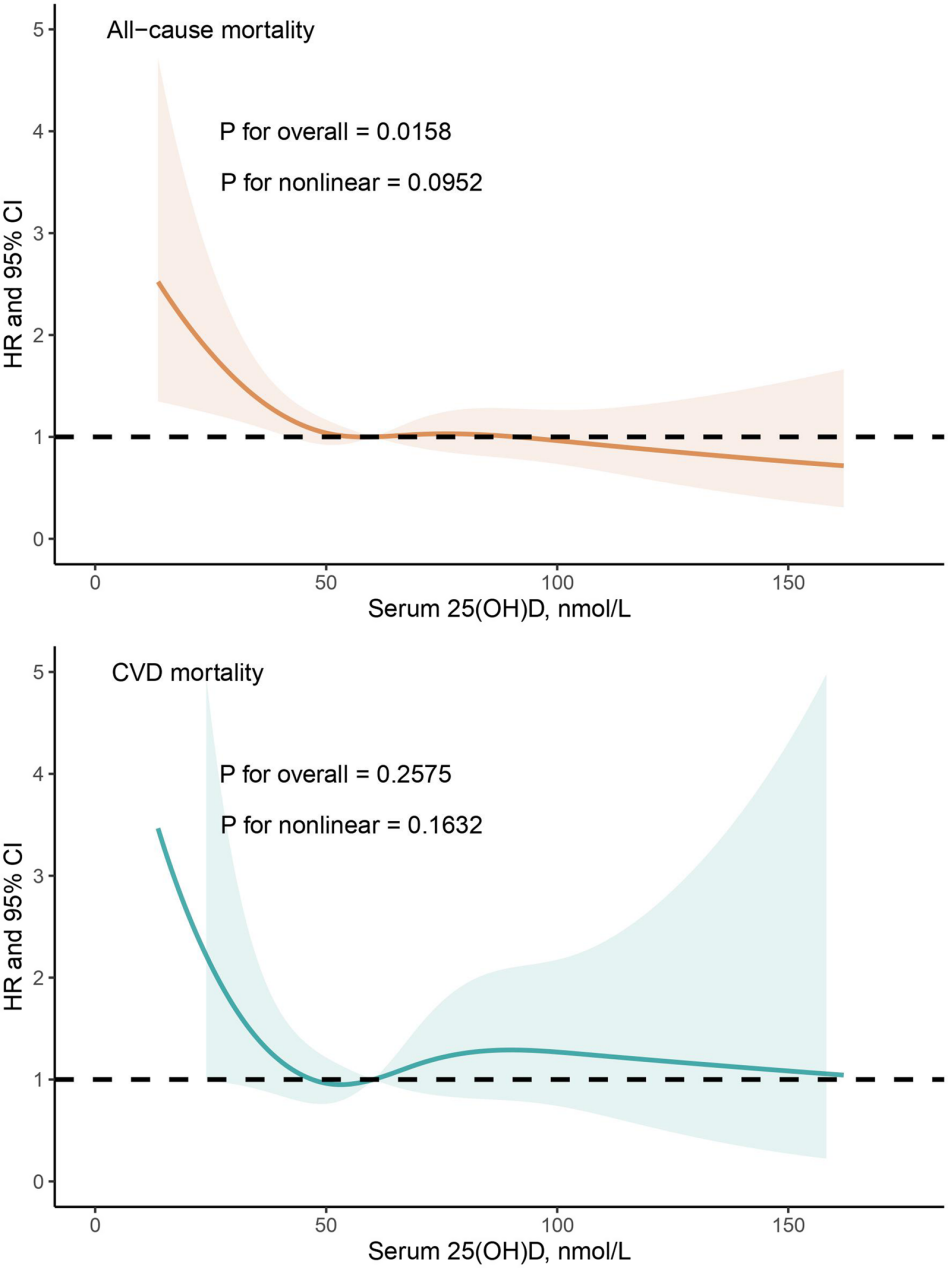
Dose–response relationship between serum 25(OH)D and the risk of all-cause death as well as cardiovascular disease (CVD) death. Restricted cubic spline model was adjusted for age, sex, ethnicity, education level, marital status, smoking status, drinking status, sedentary activity, total energy intake, sodium intake, potassium intake, overweight/obesity, vitamin D supplement use, diabetes, hypercholesterolemia, heart failure, coronary heart disease, stroke, and cancer

Figure 2 depicts the dose–response associations between serum 25(OH)D and the risk of mortality from all-cause and CVD after adjustment for multiple covariates. The restricted cubic spline model showed a linear association between serum 25(OH)D concentrations with the risk of all-cause mortality (p for overall = 0.0158, p for nonlinear = 0.0952). Nonetheless, there was no statistically significant dose–response relationship between serum 25(OH)D concentrations and the risk of CVD mortality (p for overall = 0.2575, p for nonlinear = 0.1632).

**Fig. 3:**
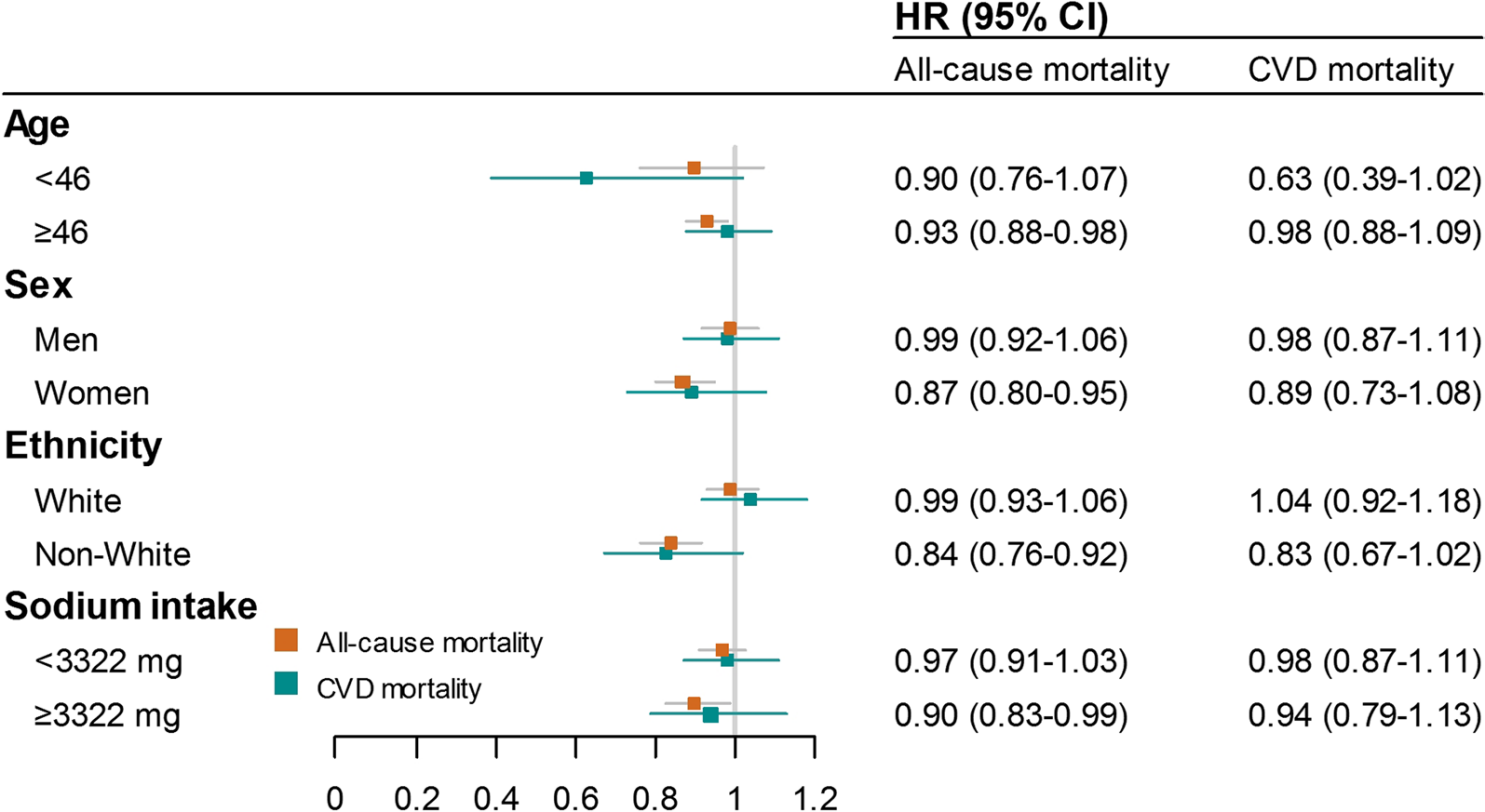
Forest plot of the association between serum 25(OH)D and the risk of all-cause death as well as cardiovascular disease (CVD) death stratified by age, sex, ethnicity, and dietary sodium intake. Hazard ratios (HRs) and 95% confidence intervals (CIs) were calculated by weighted Cox proportional hazards models with adjustment for age, sex, ethnicity, education level, marital status, smoking status, drinking status, sedentary activity, total energy intake, sodium intake, potassium intake, overweight/obesity, vitamin D supplement use, diabetes, hypercholesterolemia, heart failure, coronary heart disease, stroke, and cancer. The median age of this study population is 46 years old and the median dietary sodium intake is 3322 mg

Based on the baseline characteristics of the participants, we conducted a number subgroup analyses. When the association between serum 25(OH)D and all-cause mortality was examined, significant interactions were found between serum 25(OH)D and age, sex, ethnicity, and dietary sodium intake. We found that individuals who were older than 46 (the study population's median age), women, of non-white races, and who consumed more sodium had an significantly inverse relationship between serum 25(OH)D and the risk of all-cause death (see Fig. 3). Table S1 in the Additional file 1 displays the relationships between serum 25(OH)D concentrations and the outcomes of subgroup analysis based on all baseline characteristics.

## Discussion

In this prospective cohort study with a median follow-up of 8.8 years conducted in individuals with prehypertension, we found inverse associations of serum 25(OH)D concentrations with the risk of all-cause and CVD mortality. We also found that severe deficiency of serum 25(OH)D was associated with increased risk of all-cause and CVD mortality and this association was independent of multiple covariates including demographics, lifestyles, and several health conditions.

Epidemiological studies have demonstrated a relationship between serum 25(OH)D and the risk of CVD events and mortality in general population and patients with specific diseases. According to the Danish Copenhagen City Heart Study, plasma 25(OH)D concentration < 25 nmol/L were linked to 29% increased risk of ischemic heart disease, 49% increased risk of myocardial infarction, and 37% increased risk of early death compared those with plasma 25(OH)D concentration ≥ 75 nmol/L [21]. A meta-analysis of individual participant data of eight prospective cohort studies from Europe and the US found that comparing bottom versus top quintiles of serum 25(OH)D resulted in a pooled risk ratio and 95%CI of 1.57 (1.36–1.81) for all-cause mortality and risk ratios for cardiovascular mortality were similar in magnitude to that for all-cause mortality regardless of whether a history of CVD was present at baseline or not [22]. Several cohort studies based on the NHANES have explored the association between serum 25(OH)D concentrations and the risk of mortality in patients with diabetes [6], hyperlipidemia [7], NAFLD [8], or osteoarthritis [19]. Almost all of these studies observed an inverse association between serum 25(OH)D concentrations and all-cause mortality as well as CVD mortality. In addition, two previous cohort studies from the US NHANES and the KNHANES explored the relationship between serum 25(OH)D concentrations and mortality risk in hypertensive patients. Zhao et al. reported that serum 25(OH)D concentrations were linearly and negatively linked with the risk of mortality from all causes (p = 0.012) and from CVD (p = 0.010) among individuals with hypertension in the US based on the 2001–2004 NHANES with mortality data available through 2006 [9]. Results from the KNHANES also showed that among individuals with hypertension, those with low serum 25(OH)D status had a higher risk of cardiovascular and all-cause death [10]. Unlike hypertension, the adverse consequences of prehypertension have not received sufficient attention. Given the lack of prior studies examining the relationship between serum 25(OH)D and mortality in the prehypertensive population. In this regard, we focused on prehypertensive individuals in our study and a greater risk of all-cause and CVD mortality was observed in those who had severe serum 25(OH)D deficiency. Our study's main findings are in line with earlier research on hypertensive individuals. We also observed that those who were older, female, of non-white racial background, and who consumed higher levels of sodium in their diets had a stronger inverse relationship between serum 25(OH)D and the risk of death from all causes. This result implies that age, sex, ethnicity, and sodium intake may modify the effect of vitamin D deficiency on mortality risk; however, more research is still needed to determine specific mechanisms. Our study suggests that screening for serum 25(OH)D levels in individuals with prehypertension and supplementing vitamin D appropriately for severely deficient populations, especially the elderly, women, those of non-white races, and those with high sodium intake, may be valuable in preventing premature death.

While the exact mechanism underlying the relationship between serum 25(OH)D concentration and mortality has not yet been fully understood, experimental data indicates that vitamin D deficiency could increase the risk of cardiovascular disease and death by impacting processes like insulin resistance, inflammation, and oxidative stress [23]. Studies have demonstrated the epigenetic function of vitamin D, which impacts the transcription level of numerous genes related to insulin sensitivity, such as Insulin Receptor Substrate [24]. It has been shown that vitamin D can improve insulin resistance by increasing the capacity of healthy pancreatic β cells to secrete insulin [25]. Low grade inflammation and plasma vitamin D concentrations were found to be significantly inversely correlated [26]. Evidence from an animal study suggests that vitamin D decreases inflammation via activating the TLR-4/NF-κB signaling pathway [27]. Furthermore, by increasing cellular glutathione and antioxidant systems like glutathione peroxidase and superoxide dismutase, vitamin D may reduce oxidative stress [28]. Oxidative stress plays a crucial role in cellular injury, whereby the production of reactive oxygen species (ROS) inhibits the antioxidant defense system of the cells, eventually leading to cellular death [29]. It is widely acknowledged that oxidative stress, chronic inflammation, and insulin resistance contribute to the pathogenesis of cardiovascular disease [29, 30]. More research is necessary to further understand the mechanism by which vitamin D levels influence the risk of cardiovascular disease and death.

The limitations of our study must be taken into account when interpreting the findings. First, because cohort studies are observational in nature, a causal link between serum 25(OH)D concentrations and mortality risk cannot be established. Second, because the baseline serum 25(OH)D concentration was only assessed once, long-term trends in serum 25(OH)D concentrations could not be determined. Third, even though we took into account a number of potential confounding factors, some unmeasured or residual variables may still have an impact on the outcomes. Fourth, due to the small number of death cases, we were unable to determine if serum 25(OH)D concentrations were associated with specific types of CVD death, such as myocardial infarction, coronary heart disease, heart failure, and so on.

## Conclusions

Severe serum 25(OH)D deficiency among prehypertensive adults was associated with an increased risk of mortality from all causes as well as from CVD. Our work suggests that screening for serum 25(OH)D levels in individuals with prehypertension and supplementing vitamin D appropriately for severely deficient populations, especially the elderly, women, those of non-white races, and those with high sodium intake, may be valuable in preventing premature death.

### Supplementary Information

**Additional file 1. Table S1.** Subgroup analysis for the association between per 10 nmol/L of serum 25(OH)D increase and the risk of all-cause and CVD mortality.

## Data Availability

The datasets used and analyzed during the current study are available from the corresponding author on reasonable request.

## Abbreviations

BMI: Body mass index
CVD: Cardiovascular disease
CI: Confidence interval
DBP: Diastolic blood pressure
HR: Hazard ratio
KNHANES: Korean National Health and Nutrition Examination Survey
NHANES: National Health and Nutrition Examination Survey
RCT: Randomized clinical trial
ROS: Reactive oxygen species
SBP: Systolic blood pressure
TC: Total cholesterol
25(OH)D: 25-Hydroxyvitamin D
