# Supplementary material for: Association of serum 25-hydroxyvitamin D concentrations with all-cause and cardiovascular mortality among US adults with prehypertension: a prospective cohort study
Source: J Health Popul Nutr. 2024 Feb 6;43:24. doi: 10.1186/s41043-024-00515-5 (PMC10848370; doi:10.1186/s41043-024-00515-5)
Supplement: Supplementary file 1 — Additional file 1. Table S1. Subgroup analysis for the association between per 10 nmol/L of serum 25(OH)D increase and the risk of all-cause and CVD mortality. [file 41043_2024_515_MOESM1_ESM.docx]

**Supplemental Material**

Association of serum 25-hydroxyvitamin D concentrations with all-cause and cardiovascular mortality among US adults with prehypertension: a prospective cohort study

Yongmei Zhou, MD^#^; Yu Chen, PhD^#^; Fuli Chen, BSc; Gang Li, MD; Long Zhou, PhD

**Table S1** Subgroup analysis for the association between per 10 nmol/L of serum 25(OH)D increase and the risk of all-cause and CVD mortality^†^

|  | All-cause mortaltiy | | CVD mortality | |
| --- | --- | --- | --- | --- |
|  | HR (95%CI) | p-interaction | HR (95%CI) | p-interaction |
| Age |  | 0.0336 |  | 0.5028 |
| <46 years | 0.90 (0.76-1.07) |  | 0.63 (0.39-1.02) |  |
| ≥46 years | 0.93 (0.88-0.98) |  | 0.98 (0.88-1.09) |  |
| Sex |  | 0.0149 |  | 0.6782 |
| Men | 0.99 (0.92-1.06) |  | 0.98 (0.87-1.11) |  |
| Women | 0.87 (0.80-0.95) |  | 0.89 (0.73-1.08) |  |
| Ethnicity |  | 0.0107 |  | 0.1054 |
| White | 0.99 (0.93-1.06) |  | 1.04 (0.92-1.18) |  |
| Non-White | 0.84 (0.76-0.92) |  | 0.83 (0.67-1.02) |  |
| Education |  | 0.7803 |  | 0.2836 |
| <High school | 0.91 (0.82-1.01) |  | 0.93 (0.78-1.12) |  |
| High school | 0.98 (0.89-1.07) |  | 0.93 (0.75-1.15) |  |
| ≥High school | 0.94 (0.86-1.02) |  | 1.06 (0.90-1.26) |  |
| Smoking |  | 0.3934 |  | 0.3721 |
| Current | 0.97 (0.88-1.08) |  | 0.86 (0.65-1.16) |  |
| Former | 0.90 (0.82-0.98) |  | 1.02 (0.86-1.21) |  |
| Never | 0.96 (0.88-1.05) |  | 0.95 (0.80-1.12) |  |
| Drinking |  | 0.2703 |  | 0.0806 |
| No | 0.98 (0.89-1.08) |  | 1.01 (0.82-1.23) |  |
| Yes | 0.92 (0.87-0.98) |  | 0.92 (0.81-1.05) |  |
| Marital status |  | 0.2003 |  | 0.4008 |
| Married | 0.93 (0.87-1.00) |  | 0.95 (0.83-1.10) |  |
| Separated | 0.95 (0.87-1.04) |  | 1.01 (0.85-1.19) |  |
| Never married | 0.96 (0.82-1.13) |  | 0.94 (0.60-1.47) |  |
| Overweight/obesity |  | 0.9878 |  | 0.0539 |
| No | 0.94 (0.86-1.02) |  | 0.76 (0.61-0.94) |  |
| Yes | 0.94 (0.88-1.01) |  | 1.07 (0.95-1.21) |  |
| Sedentary hours |  | 0.1381 |  | 0.0737 |
| <5 h | 0.93 (0.85-1.02) |  | 0.93 (0.75-1.15) |  |
| ≥ 5 h | 0.95 (0.89-1.01) |  | 0.99 (0.88-1.11) |  |
| Energy intake |  | 0.1109 |  | 0.5896 |
| <2055 kcal | 0.95 (0.89-1.01) |  | 0.95 (0.84-1.07) |  |
| ≥2055 kcal | 0.92 (0.84-1.02) |  | 1.04 (0.86-1.25) |  |
| Sodium intake |  | 0.0335 |  | 0.6366 |
| <3322 mg | 0.97 (0.91-1.03) |  | 0.98 (0.87-1.11) |  |
| ≥3322 mg | 0.90 (0.83-0.99) |  | 0.94 (0.79-1.13) |  |
| Potassium intake |  | 0.7739 |  | 0.5274 |
| <2572 mg | 0.96 (0.89-1.03) |  | 0.98 (0.85-1.12) |  |
| ≥2572 mg | 0.92 (0.85-1.00) |  | 0.96 (0.81-1.14) |  |
| Comorbidities |  | 0.1792 |  | 0.0798 |
| Without | 0.96 (0.89-1.04) |  | 1.10 (0.94-1.28) |  |
| With | 0.93 (0.87-0.99) |  | 0.89 (0.77-1.03) |  |
| Vitamin D supplements use |  | 0.7314 |  | 0.8617 |
| No | 0.96 (0.89-1.03) |  | 0.97 (0.84-1.11) |  |
| Yes | 0.92 (0.85-0.99) |  | 0.93 (0.79-1.11) |  |

^†^Hazard ratios and 95% confidence intervals were calculated by weighted Cox proportional hazards model with adjustment for age, sex, ethnicity, education level, marital status, smoking status, drinking status, sedentary activity, total energy intake, sodium intake, potassium intake, overweight/obesity, vitamin D supplements use, diabetes, hypercholesterolemia, heart failure, coronary heart disease, stroke, and cancer. The grouping factor was excluded from covariates for each subgroup analysis. With comorbidities means that a person has one or more of the following five diseases: diabetes, hypercholesterolemia, coronary heart disease, stroke or cancer. The median values of age, sedentary hours, total energy intake, dietary sodium intake, and dietary potassium intake were 46 years, 5 h, 2055 kcal, 3322 mg, and 2572 mg, respectively. 25(OH)D, 25-hydroxyvitamin D.
